# MicroRNAs in the axon and presynaptic nerve terminal

**DOI:** 10.3389/fncel.2013.00126

**Published:** 2013-08-06

**Authors:** Barry B. Kaplan, Amar N. Kar, Anthony E. Gioio, Armaz Aschrafi

**Affiliations:** ^1^Laboratory of Molecular Biology, National Institute of Mental Health, National Institutes of Health, BethesdaMD, USA; ^2^Department of Cognitive Neuroscience, Donders Institute for Brain, Cognition, and Behavior, Radboud University Nijmegen Medical CenterNijmegen, Netherlands

**Keywords:** axonal mRNAs, mitochondrial mRNAs, intra-axonal protein synthesis, translational regulation, energy metabolism, ROS generation, axonal growth

## Abstract

The distal structural/functional domains of the neuron, to include the axon and presynaptic nerve terminal, contain a large, heterogeneous population of mRNAs and an active protein synthetic system. These local components of the genetic expression machinery play a critical role in the development, function, and long-term viability of the neuron. In addition to the local mRNA populations these presynaptic domains contain a significant number of non-coding RNAs that regulate gene expression post-transcriptionally. Here, we review a small, but rapidly evolving literature on the composition and function of microRNAs that regulate gene expression locally in the axon and nerve terminal. In this capacity, these small regulatory RNAs have a profound effect on axonal protein synthesis, local energy metabolism, and the modulation of axonal outgrowth and branching.

## INTRODUCTION

The distal structural/functional domains of the neuron (i.e., axon, presynaptic nerve terminal, and dendrite) are well known to contain a highly diverse population of mRNAs and an active protein synthetic system. In large, asymmetric neurons, these basic components of the genetic machinery play an especially important role in the development and maintenance of its cellular polarity, as well as in its synaptic plasticity, regeneration, and repair (for review, see Jung et al., 2012).

Initial estimates of the diversity of these mRNA populations, as derived from invertebrate model systems, suggested the presence of 200–400 different mRNAs (Perrone-Capano et al., 1987; Moccia et al., 2003). However, the study of mammalian neurons and the resolution afforded by advanced gene profiling methodology has revealed a remarkable complexity in the axonal transcriptome (Willis et al., 2005; Taylor et al., 2009; Zivraj et al., 2010; Gumy et al., 2011). These messengers encode a complex set of proteins that can be organized into several functional categories to include: cytoskeletal and scaffolding proteins, translation factors and ribosomal proteins, molecular motors and chaperones, and metabolic enzymes.

In addition to these diverse mRNA populations, axons and nerve terminals also contain numerous microRNAs (miRNAs). These highly conserved, small, non-coding RNAs play a key role in the post-transcriptional regulation of gene expression. In general the bio-genesis and function of miRNAs in the nervous system has been well reviewed (Schratt, 2009; Siegel et al., 2011; Olde Loohuis et al., 2012). In this brief communication, attention will be focused on those miRNAs that function to post-transcriptionally regulate gene expression locally in the distal structural domains of the neuron.

## IDENTIFICATION OF miRNAs IN THE AXON

In a recent study, Natera-Naranjo et al. (2010) employed primary sympathetic neurons cultured in compartmentalized Campenot chambers to obtain a pure axonal RNA fraction to identify the component miRNAs by microarray analysis. Surprisingly, this study revealed considerable complexity in the miRNA population present in this cellular compartment. The relative abundance of several of these miRNAs was found to be highly enriched in the axon as compared to the parental cell bodies, a finding that raised the possibility that there could be a selective transport of these molecules into the axon (see below).

Of course, estimation of the number of miRNAs present in the axon, as derived from microarray profiling, is totally dependent upon the numerical threshold used to determine a positive signal (i.e., the signal to noise ratio). To address this issue, the authors employed the convergence of the microarray data with the results obtained from quantitative RT-PCR analyses. High correlations between the microarray data and the results of quantitative RT-PCR were obtained for miRNAs with microarray signal intensities greater than one standard deviation above median background values. Hence, using this as a “cut-off” value it was determined that there was approximately 137 different miRNAs in the axons of rat superior cervical ganglion neurons (Natera-Naranjo et al., 2010). It bears mention here that the cell culture system used in these studies precludes one from distinguishing between the axon and nerve terminal, and hence reference to these two cellular compartments will be combined throughout the review.

Bioinformatic search for putative mRNA targets of the axonally abundant miRNAs revealed an enrichment of transcripts that encode proteins that function in neuronal signaling, mRNA and protein transport, as well as, mRNA transcription and translation. Interestingly, this analysis also identified a subset of axonal miRNAs that potentially target multiple mRNAs involved in related cellular functions. These observations suggest that these small, non-coding RNAs in the axon could function to integrate/orchestrate multiple functions in the axon.

Many miRNAs are expressed as clusters on a single polycistronic transcript. Interestingly, the relative abundance of some miRNAs derived from polycistronic transcripts differ markedly in the axon as compared to their parental cell soma (Natera-Naranjo et al., 2010). For example, the miRNA-17-92 cluster is comprised of six individual miRNAs. The relative abundance of two of the mature miRNAs were two- to fourfold greater in the axon than in the corresponding cell bodies (Natera-Naranjo et al., 2010; Zhang et al., 2013). After over-expression of the miRNA-17-92 cluster in embryonic cortical neurons cultured in microfluidity chambers, the relative abundance of miR-19b and miR-20a, two components of the polycistronic transcript, were approximately 5- to 20-fold greater in the axon compared to the cell soma (Zhang et al., 2013). The mechanism underlying the differential or selective transport of these miRNAs is currently unknown, but is eminently worthy of future investigation.

## ACTIVITY OF THE AXONAL PROTEIN SYNTHETIC SYSTEM IS MODULATED BY miRNAs

One of the most abundant miRNAs present in the axons of sympathetic neurons is miR-16. Results of a bioinformatics search for mRNAs whose 3′untranslated regions (UTRs) contained miR-16 binding sites revealed two mRNAs that encoded eukaryotic translation initiation factors, eIF2B2 and eIF4G2 (Kar et al., 2013). The cognate mRNAs for both these factors are present in the axon and the local expression of eIF2B2 and eIF4G2 proteins can be modulated by miR-16. The regulation of the expression of these factors was shown to be effected through the binding of miR-16 to the 3′UTR with subsequent degradation of the mRNAs. The transfection of the precursor miRNA directly into the axon greatly reduced the levels of these factors and markedly inhibited the activity of the local protein synthetic system, as judged by metabolic labeling studies. Similar effects on local protein synthesis and axon growth were observed after small interfering RNA-mediated knockdown of axonal eIF2B2 and eIF4G2 mRNA. Taken together, these findings demonstrated that the expression of miRNAs in the distal axon could modulate local protein synthesis by regulating the expression of key components of the translation system.

## miRNAs REGULATE ENERGY METABOLISM IN THE AXON

One surprising feature of all axons studied to date is the presence of a large number of nuclear-encoded mitochondrial mRNAs. It has been estimated that in large invertebrate axons and presynaptic nerve terminal nearly one-quarter of the newly synthesized protein is destined for mitochondria, and that the membrane potential and activity of this organelle is highly dependent on the local synthesis of these rapidly turning over proteins (Hillefors et al., 2007; Kaplan et al., 2009).

**FIGURE 1:**
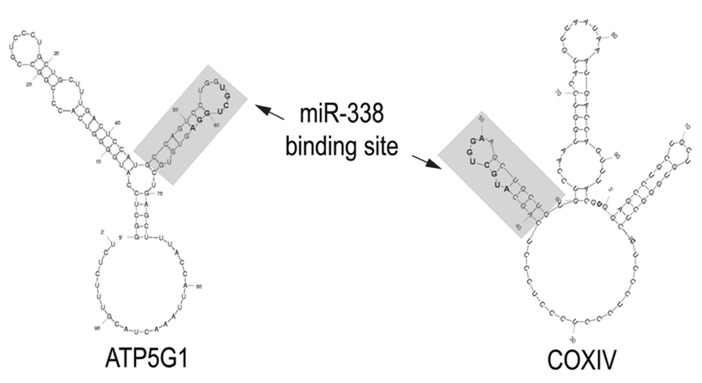
**miR-338 binding sites in nuclear-encoded mitochondrial mRNAs present in the axon.** The secondary structure of the 3′UTRs of CoxIV and ATP5G1 mRNAs were predicted by Mfold analyses. The miR-338 binding site present in a 38-nucleotide stem-loop structure is indicated in gray. Nucleotides comprising the microRNA targeting sequence are bolded. Modified from Aschrafi et al. (2012).

It is noteworthy that several of these nuclear-encoded mitochondrial mRNAs code for proteins that play a key role in oxidative phosphorylation, a finding that suggests that their local synthesis might contribute to the regulation of ATP production. At least two of these mitochondrial mRNAs, cytochrome c-oxidase (CoxIV) and ATP synthase (ATP5G1) contain a binding site for miR-338 in their 3′UTRs. Both of these binding sites are situated in a hairpin-loop structure that could facilitate miRNA accessibility (**Figure 1**). Axonal transfection studies conducted with chimeric reporter gene constructs containing these putative binding sites established that they were *bona fide *targets of miR-338 (Aschrafi et al., 2008, 2012). Consistent with these findings, the over-expression of miR-338 in the axon greatly reduced the levels of endogenous CoxIV and ATP5G1 mRNA and protein in the axon, and resulted in marked reduction in local ATP levels and elevation in the production of reactive oxygen species (ROS). In contrast, inhibition of endogenous miR-338 function had the opposite effects on axonal ATP levels and ROS generation.

To delineate the impact of the modulation of local levels of miR-338 on the metabolic rate and function of axons of noradrenergic sympathetic neurons, mitochondrial oxygen consumption, as estimated by reduction of the redox dye Alamar Blue, as well as norepinephrine uptake into the axon was assessed after introducing precursor miR-338 or anti-miR-338 directly into the axon. Under the cell culture conditions employed in these experiments, modulation of the local levels of miR-338 had a profound effect on the metabolic rate and function of the axon (Aschrafi et al., 2008).

## LOCAL EXPRESSION OF miRNAs EFFECT AXONAL GROWTH AND BRANCHING

In light of the fact that miRNAs can influence the activity of the intra-axonal protein synthetic system, as well as local energy metabolism, it is not surprising that the expression of these small regulatory RNAs could have profound effects on the growth and branching of the axon. For example, elevation of the levels of miR-338 or miR-16 in the axons of cultured primary sympathetic neurons inhibits their rate of elongation (Aschrafi et al., 2008, 2012; Kar et al., 2013). The attenuation in axonal outgrowth could be attributed, at least in part, to the dysregulation of mitochondrial function with consequent elevation in the production of ROS in the axon. In this regard, partial restoration of axonal growth could be effected by the application of anti-oxidants to the culture media (Natera-Naranjo et al., 2012).

In a recent study, Zhang et al. (2013) reported the expression of the components of the miR-17-92 cluster in the distal axons of primary embryonic cortical neurons. Over-expression of this cluster substantially increased axonal outgrowth, whereas the inhibition of endogenous miR-19a, a key component of the cluster, suppressed axonal growth. The local effects of this miRNA were attributed to the modulation of phosphatase and tension homolog (PTEN) protein levels by miR-19a.

In an elegant series of experiments, Dajas-Bailador et al. (2012) demonstrated that inhibition of local miR-9 in primary embryonic cortical neurons facilitated axonal outgrowth and inhibited branching of the axon. Ostensibly, these effects were mediated through the regulation of one of its targets, microtubule-associated protein 1b (MAP1B). Interestingly, although miR-9 was detected in dendrites, the over-expression of this miRNA had no effect on their length, a finding which could reflect the preferential localization of Map1b mRNA to the developing axon.

To assess the role of miR-9 *in vivo*, Dajas-Bailador et al. (2012) introduced a specific miR-9 inhibitor into the cerebral cortex of 14.5 day mouse embryos. The inhibition of endogenous miR-9 activity resulted in a severe disruption of neuronal migration and differentiation.

Taken together, these findings indicate that miRNAs play an important role in the function and development of the axon through the post-transcriptional modulation of the expression of key constituents of the local mRNA population.

## DISCUSSION

Recent research has revealed the presence of a diverse population of miRNAs in the axon and presynaptic nerve terminal. Nonetheless, only a few of these miRNAs and their target genes have been characterized, and hence the function of the vast majority of these non-coding RNAs remains unknown. Some of these miRNAs are expressed specifically in brain and the relative abundance of others differs markedly in the various cellular compartments of the neuron. These observations suggest a unique regulatory role for these RNAs in the development, maintenance and function of the distal regions of this highly polarized cell.

One remarkable feature of miRNAs is that they can co-ordinately regulate the expression of multiple mRNAs that encode proteins with related cellular functions. In this regard, miRNAs might be envisaged as “master regulators” of the expression of batteries of genes which comprise functional networks and/or pathways (for review, see Olde Loohuis et al., 2012). One case in point, is miR-338 which targets several mRNAs that code for proteins that are key components of the enzymatic complexes that comprise the oxidative phosphorylation chain. It is important to note here, that the effects of miR-338 on CoxIV and ATP5G1 expression are additive and hence, this miRNA could serve to “fine-tune” the local production of ATP in response to neuronal activity.

**FIGURE 2:**
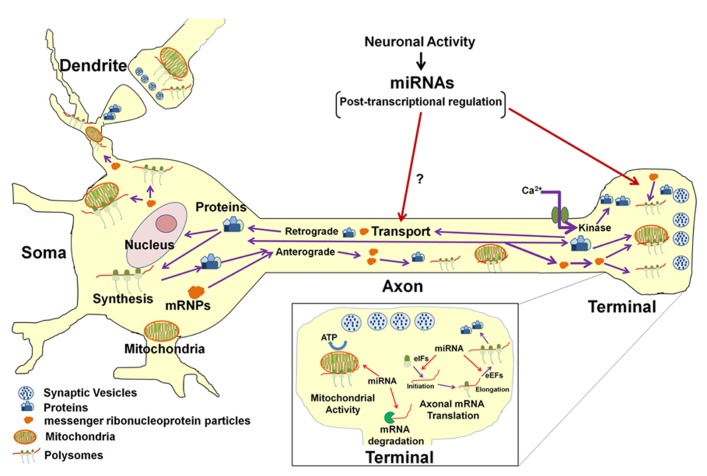
**Model for miRNA-mediated regulation of neuronal function.** In the neuron, protein synthesis occurs in multiple compartments that include the cell body, dendrite, axon, and presynaptic nerve terminal. A subset of mRNAs transcribed in the nucleus are packaged into stable messenger ribonucleoproteins complexes (mRNPs) and are selectively and rapidly transported to the distal structural/functional domains of the neuron. The selective translation of these localized mRNAs plays key roles in neuronal development, axon growth and maintenance, and synaptic plasticity. Neuronal miRNAs function at multiple levels within the neuronal gene expression system to modulate neuronal activity and function. It has been shown that miRNAs regulate the local post-transcriptional gene expression of specific target mRNAs that encode factors affecting mitochondrial activity (see inset) as well as axonal growth and branching. In addition, miRNAs can also modulate translation of multiple mRNAs in the axon and nerve terminal by regulating local expression of eukaryotic translation factors (see inset). It is also conceivable that miRNAs might regulate the local synthesis and retrograde transport of transcription factors in response to growth factors or neural injury and hence influence gene transcription in the parental soma. Last, miRNA control of the local synthesis of cytoskeletal and/or motor proteins might facilitate the regulation of their own anterograde transport to their ultimate sites of function.

On a more global level, non-coding RNAs, such as miR-16, have been shown to regulate the activity of the axonal protein synthetic system, itself. This effect was mediated through the modulation of the expression of two translation initiation factors (**Figure 2**). Thus, through this mechanism, miR-16 could affect the local expression of large numbers of gene products. One might speculate that some of these locally synthesized proteins are likely to play an important role in the activity-dependent modulation of the function of the axon and nerve terminal, as well as in synaptic plasticity.

In view of the effects of miRNA on protein synthesis and local energy metabolism, it is not surprising that these non-coding RNAs could have a profound effect on the growth and development of the axon. These findings support the hypothesis that miRNAs play a central role in gene regulatory networks involved in axon development, as well as the plasticity of the presynaptic nerve terminal. These observations also raise the possibility that dysregulation of miRNA function might play a role in the pathophysiology of neurological and psychiatric disorders (for review, see Qureshi and Mehler, 2012; Rege et al., 2013).

Despite the evidence for the existence of a host of miRNAs in the axon, there is a dearth of knowledge regarding the mechanism(s) underlying the transport of these RNAs to the distal regions of the neuron. Several models have been proposed for the selective shuttling of miRNAs to the synapse (for example, see Kosik, 2006). These working hypotheses include: co-delivery of the miRNAs with their cognate mRNA targets via RNA granules; mRNA-independent delivery of mature/functional miRNAs; and the delivery of precursor miRNAs followed by their sequential local processing to the mature, functional form of the non-coding RNA. This last model is supported by several reports of the presence of miRNA processing machinery in mammalian axons (Hengst et al., 2006; Aschrafi et al., 2008; Zhang et al., 2013). In addition, the introduction of precursor miRNAs directly into the axon results in a marked increase in the levels of the mature, functional forms of the molecule within hours (Aschrafi et al., 2008). These observations clearly indicate that axons have the capability to process precursor miRNAs to mature forms of the molecule. One interesting question for future research is whether synaptic activity could regulate axonal miRNA trafficking or alternatively regulate the retrograde transport of protein(s) that could influence miRNA transcription in their parental neurons (**Figure 2**). In this regard, it is well known that transcription factors can be locally synthesized in the axon and retrogradely transported to the neuronal cell nucleus, especially in response to neurotrophic signaling and/ or neuronal injury (Cox et al., 2008; Gumy et al., 2010; Ben-Yaakov et al., 2012). However, to date, no studies have investigated the role that miRNAs might play in the local regulation of axon regeneration.

Future characterization of the axonal miRNA regulatory landscape will generate further insights into the molecular basis of axonal activity, maintenance, and neuronal function. Moreover, recent studies have delineated the functional significance of miRNAs in the regulation of local translation in developing axons, dysregulation of which might ultimately underlie the etiology of neurodevelopmental disorders and mental illness.

## Conflict of Interest Statement

The authors declare that the research was conducted in the absence of any commercial or financial relationships that could be construed as a potential conflict of interest.
